# Broadband and Enhanced Second‐Harmonic Generation in Metallic 3R‐TaS_2_

**DOI:** 10.1002/smll.202513532

**Published:** 2026-08-14

**Authors:** Ruo‐Si Chen, Chuanyu Wang, Xiangqi Liu, Zhuoyuan Lu, Shuyao Qiu, Boqing Liu, Tieyu Lü, Shi‐Rui Zhang, Yanfeng Guo, Yuerui Lu

**Affiliations:** ^1^ School of Engineering College of Systems and Society Australian National University Canberra Australia; ^2^ ShanghaiTech Laboratory for Topological Physics ShanghaiTech University Shanghai China; ^3^ Department of Physics and Institute of Theoretical Physics and Astrophysics Xiamen University Xiamen China; ^4^ Research School of Physics Department of Electronic Materials Engineering Australian National University Canberra Australia

**Keywords:** broadband nonlinear optics, metallic 3R‐TaS_2_, second‐harmonic generation, SHG enhancement, van der Waals systems

## Abstract

Second‐harmonic generation (SHG) serves as a fundamental mechanism for frequency conversion and light manipulation in photonic devices, as well as a powerful probe for exploring nonlinear optical properties in 2D materials. However, in metallic 2D systems, strong free‐carrier absorption and shallow optical penetration depth usually suppress the nonlinear response. Realizing pronounced SHG in such highly absorptive materials thus remains a major challenge in nonlinear photonics. In this work, we systematically investigate the SHG response of non‐centrosymmetric metallic 3R‐TaS_2_ and demonstrate strong broadband SHG across a wide emission wavelength range of 510–705 nm. By analyzing the wavelength‐dependent optical constants and dielectric function, we show that this broadband SHG arises from the combined effects of the non‐centrosymmetric crystal structure, a dielectric‐like optical response in the visible range, and the absence of sharp resonance features, together with free‐carrier‐mediated optical damping. Furthermore, substantial SHG enhancement is achieved using a reflective gold substrate, with the enhancement factor increasing from 2.7 to 45.5 as the flake thickness decreases from 123 to 68 nm. The extracted effective second‐order nonlinear susceptibility is comparable to that of MoS_2_, despite the metallic electronic structure of 3R‐TaS_2_. These results demonstrate that strong, broadband nonlinear optical responses can persist in metallic layered materials and provide new insights into the interplay among metallicity, optical response, and nonlinear light‐matter interactions in van der Waals systems.

## Graphical abstract

A pronounced and robust SHG response has been observed in metallic 3R‐TaS_2_. It shows broadband SH conversion from 1020 nm to 1410 nm. Moreover, compared to the sapphire substrate, 3R‐TaS_2_ flakes with various thicknesses on the gold substrate showed different SHG enhancement; the enhancement factors range from 2.7 to 45.5.

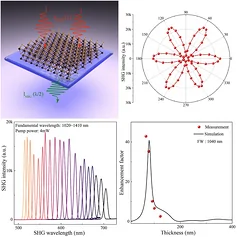

## 1 Introduction

Second harmonic generation (SHG) is a fundamental nonlinear process that is widely applied in non‐classical light source generation [1] and biological imaging [2]. Generally, SHG occurs in crystals with intrinsic broken inversion symmetric nature, such as lithium niobate (LiNbO_3_) [3], beta barium borate (BBO) [4], and so on. However, conventional nonlinear crystals are always with bulk thickness, which limits the development of on‐chip nonlinear nanophotonics. To meet the requirement of downscaling nonlinear optical devices, exploring low‐dimensional materials with noncentrosymmetric structure and large second‐order susceptibility (*χ^(2^* ^)^) is of great importance for the development of next‐generation photonic circuits. The discovery of 2D materials provides new opportunities for miniaturization of nonlinear optical (NLO) devices by reason of the atomic‐level thickness.

2D layered materials have attracted significant interest due to their unique structural and optical properties, particularly their strong in‐plane covalent bonding and weak interlayer van der Waals interactions, which enable facile mechanical exfoliation into ultrathin flakes. Over the past decades, extensive studies have been devoted to the nonlinear optical properties of 2D transition metal dichalcogenides (TMDs), such as monolayer MoS_2_ [5], MoSe_2_ [6], WS_2_ [7], WSe_2_ [8], and 3R‐phase TMDs [9, 10]. However, most reported 2D nonlinear materials are semiconductors or insulators [11, 12], whereas 2D metallic nonlinear materials remain largely unexplored.

In metallic systems, second‐order nonlinear responses are typically suppressed due to strong screening by free carriers, leading to weak or negligible SHG [13]. Nevertheless, recent discoveries in Weyl semimetals, such as β‐WP_2_ and Td‐WTe_2_, have demonstrated unexpectedly strong SHG arising from their topological electronic structures and Berry curvature effects [14, 15, 16, 17]. Although these findings highlight the potential of metallic systems for nonlinear optics, they are mainly limited to bulk materials. In 2D metallic systems, such as graphene, SHG can only be induced when inversion symmetry is intentionally broken by external perturbations, including non‐uniform strain [18], in‐plane optical fields [19], or external electrical bias [20]. Therefore, the exploration of intrinsic 2D metallic nonlinear materials is highly important for advancing compact, high‐performance photonic and quantum devices.

2D tantalum disulfide (TaS_2_), as a member of 2D TMDs, was demonstrated as a layered metallic conductor in the 1970s [21]. It has aroused tremendous interest due to its special electrical properties, such as unconventional superconductivity [22, 23, 24], rich charge‐density‐wave (CDW) phases [25, 26], etc. Therefore, 2D TaS_2_ in different polytypes (1T, 2H, and 3R) has been widely applied as an efficient catalyst in the hydrogen evolution reaction (HER) due to its high electrical conductivity [27, 28, 29]. Nevertheless, the previous research of TaS_2_ mainly focused on the phase transition of CDW, superconductivity, and HER; the nonlinear optical properties of 2D TaS_2_ have never been explored. It should be noted that 2D TMD materials in their 3R phase are intrinsically noncentrosymmetric, which is the fundamental physical condition that enables SHG. Thus, 3R TaS_2,_ as an intrinsic nonlinear material, is one of the most suitable research candidates in 2D metallic nonlinear systems.

Despite exploring more novel 2D nonlinear materials, modulation of SHG response is also a key research field for the future development of nonlinear optical devices. In order to enhance the SHG intensity of 2D materials, efforts have been made to modify the nonlinear effects in the past few years. For example, quasi‐phase‐matching by twisted stacking of 3R‐MoS_2_ have achieved enhanced SHG intensity with an enhancement factor of 7.5 by Tang et al. [9, 30]. Moreover, ultrathin 3R‐MoS_2_ metasurfaces have been proposed to enhance the SHG intensity of 3 orders of magnitude [31, 32]. However, the mechanisms governing SHG modulation in metallic layered materials remain largely unexplored. In particular, whether broadband SHG can be maintained in metallic systems and how substrate‐induced local‐field effects interact with free‐carrier‐mediated optical responses are still open questions. Addressing these issues is important not only for understanding nonlinear light–matter interactions in metallic van der Waals materials, but also for expanding the available material platforms for nonlinear photonics.

In this study, we systematically investigate the second‐order nonlinear optical response of metallic 3R‐TaS_2_ using wavelength‐ and polarization‐resolved SHG measurements. Remarkably, strong and robust SHG is observed despite the presence of free carriers and optical absorption, which are typically associated with metallic systems. The SHG response is further examined as a function of excitation power, wavelength, and flake thickness. The results reveal broadband SHG conversion over a wide fundamental wavelength range of 1020–1410 nm, corresponding to SHG emission from 510 to 705 nm, together with pronounced thickness‐dependent nonlinear optical behavior. Analysis of the optical constants and dielectric function indicates that the broadband SHG originates from the combined effects of the non‐centrosymmetric crystal structure, dielectric‐like optical response in the visible spectral range, and the absence of sharp resonances. Furthermore, the SHG intensity can be significantly enhanced by a reflective gold substrate through local‐field enhancement and interface‐mediated optical effects. Compared with sapphire substrates, enhancement factors of 2.7, 6.6, 10.3, 37.3, and 45.5 are obtained for 3R‐TaS_2_ flakes with thicknesses of 123, 100, 92, 79, and 68 nm, respectively. The extracted effective second‐order susceptibility is comparable to that of MoS_2_ despite the metallic electronic structure of 3R‐TaS_2_. These findings demonstrate that strong and broadband second‐order nonlinear optical responses can persist in metallic layered materials, providing new insights into the interplay among metallicity, optical response, and nonlinear light‐matter interactions, while offering a promising platform for nonlinear photonics based on metallic van der Waals crystals.

## 2 Results and Discussion

### 2.1 SHG Performance of 3R‐TaS_2_

**FIGURE 1:**
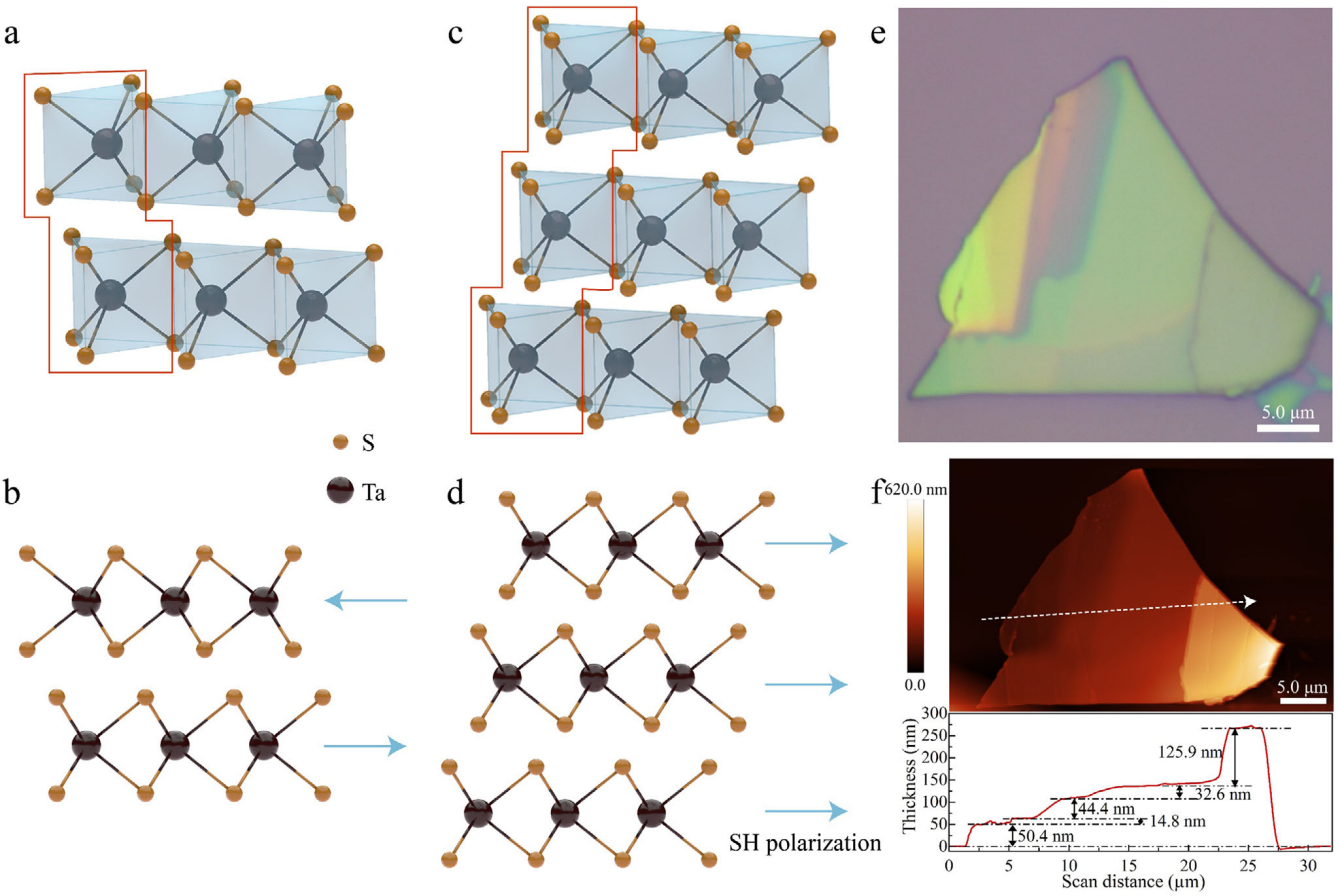
Crystal structure and characterization. a) Schematic of the crystal structure of 2H‐TaS_2_. The unit cells with inverse orientation in adjacent layers are outlined in red. b) Side view of the atomic structure of 2H‐TaS_2_. The adjacent atomic layers show flipped orientation, corresponding to the anti‐parallel direction of the SH dipoles that allows for SHG. c) Schematic of the crystal structure of 3R‐TaS_2_. The individual layer is identical to 2H‐TaS_2_. However, the unit cells in adjacent layers have the same orientation (outlined in red). d) Side view of atomic structure of 3R‐TaS_2_. All layers are oriented in the same direction, enabling the constructive interference of the SHG response due to the parallel dipoles. e) Optical image of 3R‐TaS_2_ on 285 nm SiO_2_/Si substrate. 3R‐TaS_2_ with different thicknesses can be identified through the color difference of the materials, which is caused by the interference effects. f) Atomic force microscopic (AFM) image of 3R‐TaS_2_. The height profile plotted at the bottom is extracted along the white dashed line in the upper image.

Figure 1 gives the schematic diagrams of the atomic structures of TaS_2_ in its hexagonal (2H) and 3R phases. The crystal structure of 2H‐TaS_2_ is shown in Figure 1a. Each Ta atom is surrounded by six S atoms, forming a trigonal prismatic coordination environment. The bulk 2H phase belongs to the hexagonal *P6_3_/mmc* space group with *D_6h_* point‐group symmetry. The side view of 2H‐TaS_2_ is presented in Figure 1b. In 2H‐TaS_2_, adjacent S–Ta–S layers are stacked with a 180° in‐plane rotation relative to each other, leading to inversion‐related layer orientations and an overall centrosymmetric structure. The centrosymmetric nature indicates that 2H‐TaS_2_ with even‐numbered layers would exhibit no SHG signal because of the opposite layer polarities along the out‐of‐plane direction, leading to the cancellation of the SH polarization in an antiparallel stacking configuration between adjacent layers. However, measurable SHG would appear in odd layers due to the broken centrosymmetric nature [33]. Figure 1c, d schematically show that the S‐Ta‐S trilayers in 3R‐TaS_2_ are stacked in the same orientation. This stacking sequence breaks inversion symmetry and is consistent with the non‐centrosymmetric *R3m* space group, corresponding to the *C_3__v_* point group. The parallel dipoles in the same direction lead to constructive interference of SHG polarization from adjacent layers (e.g., bilayer at N═2), thereby enabling the N^2^ scaling enhancement of the SHG response in the 2D limit [34]. Figure 1e denotes an optical microscopy image of a specific 3R‐TaS_2,_ which presents an intuitive view of contrast with the SiO_2_/Si substrate for different thicknesses. The atomic force microscopic (AFM) image of the representative sample is illustrated in Figure 1f. The height profile at the bottom of Figure 1f is extracted along the white dashed line from the AFM image. Raman spectroscopy under the excitation wavelength of 532 nm is performed to analyze the optical properties of the exfoliated 3R‐TaS_2_ (Note S1 and Figure S1). The Raman spectrum shows good consistency with previous findings [35], in which the Raman peaks of the in‐plane vibration ($E_{2g}^{1}$) and out‐of‐plane vibration (*A*_1*g*_) modes are at 291 and 395 cm^−1^, respectively.

To demonstrate the metallic properties of 3R‐TaS_2_, we provide the band structure and density of states in Note S2 (Figure S2). The calculated band structure in Figure S2a without spin–orbit coupling shows several bands crossing the Fermi level (*E_F_*), accompanied by a nonzero density of states at *E_F_* (Figure S2b), suggesting metallic or semimetallic electronic behavior of 3R‐TaS_2_. It should be noted that this electronic metallicity does not necessarily imply a strongly metallic optical response over the entire spectral range. Therefore, the DFT results mainly provide evidence for the existence of free carriers and the associated Drude contribution, while the wavelength‐dependent optical response is determined by the frequency‐dependent dielectric function. All first‐principles calculations were carried out within the framework of density functional theory (DFT) using the projector augmented‐wave (PAW) method, as implemented in the Vienna Ab initio Simulation Package (VASP) [36]. The exchange–correlation interaction was treated using the generalized gradient approximation (GGA) in the Perdew–Burke–Ernzerhof (PBE) form [37]. Long‐range van der Waals interactions were accounted for using the DFT‐D3 correction scheme [38]. A plane‐wave cutoff energy of 500 eV was used for all calculations. The electronic self‐consistent field convergence criterion was set to 10^−5^ eV. The Brillouin zone was sampled using a 6 × 6 × 3 Monkhorst–Pack k‐point mesh. All structures were fully relaxed until the residual force on the atoms had declined to less than 0.02 eV/Å. The electronic band structure was calculated along the high‐symmetry path Γ–M–K–Γ–A–L–H–A, and the Fermi level was set to 0 eV. Spin–orbit coupling was not included in the present calculations.

**FIGURE 2:**
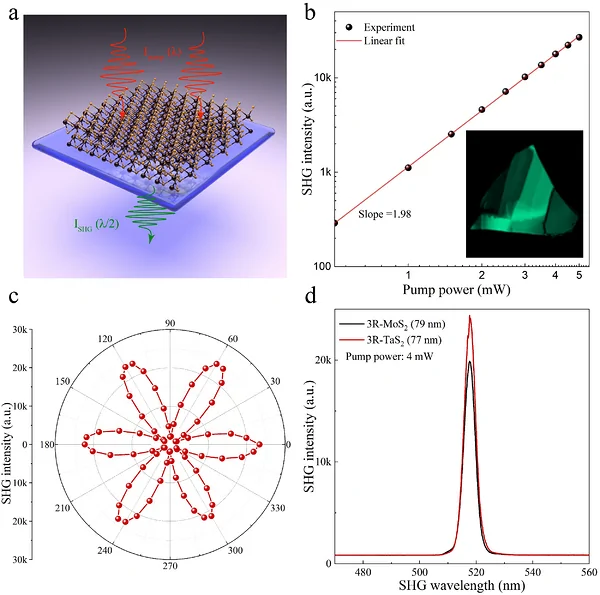
SHG measurement of 3R‐TaS_2_. a) Schematic illustration of SHG measured at transmission mode from 3R‐TaS_2_. The linearly polarized pump reaches the front surface of 3R‐TaS_2_, then passes through the sample and the sapphire substrate, and the SHG emission light is detected from the back surface of the sapphire substrate. b) Measured SHG intensity as a function of the pump power from a specific 3R‐TaS_2_ flake with a thickness of 67 nm (black dots). The extracted power law index of the linear fitting function (red line) is 1.98. Inset: SHG mapping of the 3R‐TaS_2_. c) Polarization‐dependent SHG intensity for 3R‐TaS_2_. d) SHG intensity of 3R‐TaS_2_ employed in this work and a 3R‐MoS_2_ with similar thickness as a reference. The FW is 1040 nm, and the average excitation power is 4 mW.

The schematic illustration of SHG emission in transmission mode is displayed in Figure 2a. The linearly polarized laser with a fundamental wavelength (FW) of λ reaches the front side of the 3R‐TaS_2_ and passes through the material, and the SHG signal with a wavelength of λ/2 is collected from the back surface of the sapphire substrate. The FW in our experiment ranges from 1020 to 1410 nm. The SHG measurements of the incident power, polarization, wavelength, and thickness‐dependent SHG in this study are all conducted at transmission mode. Herein, a Mai Tai femtosecond (fs) laser combined with an optical parametric oscillator (OPO) was used as the fundamental laser source. The laser operates at a repetition rate of 80 MHz with a pulse duration of approximately 100 fs. The excitation beam was focused onto the sample with a spot diameter of ∼7 µm at the focal plane. The average excitation power of the incident laser is generally kept stable at 4 mW in this study, which is measured by a power meter with an accuracy of the nW level, allowing the precise control of incident laser power. Detailed information of SHG measurement and setup can be found in the Experimental Section and Note S3 (Figure S3). Figure 2b displays the logarithmic plot of the SHG peak intensity as a function of excitation power. It can be observed that the emission intensity is positively related to the incident laser power. The slope extracted from the experimental data is 1.98, which is close to the value of 2 calculated from the electric dipole theory. The result indicates a quadratic relationship between the excitation power and the SHG intensity, consistent with the SHG principle [39]. The inset of Figure 2b shows the image of SHG mapping of a 3R‐TaS_2_. In addition, we performed repeated measurements over the same power range (5 mW) and did not observe any irreversible change in the SHG intensity, indicating that the sample remains stable under the applied excitation conditions, as can be seen from Figure S4 (Note S4). Besides, the 3R‐TaS_2_ also shows good stability in the ambient environment. As shown in Figure S5 (Note S5), we have performed long‐term SHG stability measurements on the same 3R‐TaS_2_ samples after exposure to the ambient environment for different periods of time. The SHG intensity of the samples with different thicknesses shows no observable degradation over 1 month. These results indicate that the nonlinear optical response of the studied 3R‐TaS_2_ flakes is robust against ambient air exposure, suggesting that any possible surface oxidation does not significantly affect the bulk‐dominated SHG signal under our experimental conditions. Although we cannot completely exclude the possibility of subtle surface modifications, such as the formation of an ultrathin oxide layer, the negligible variation in SHG response suggests that any such surface changes have a limited influence on the overall optical properties and conclusions of the present work. We further conducted polarization‐dependent SHG to investigate the second‐order nonlinear properties of 3R‐TaS_2_, as plotted in Figure 2c. The 6‐fold symmetry in the polar plot as a function of the polarization angle *θ* originates from the in‐plane crystal symmetry (*C_3v_*). The measured polarization‐resolved SHG of 3R‐TaS_2_ can be described by *I* ═ *I*_0_ *cos* ^2^(3θ) under the co‐polarization configuration, where the polarization orientation of the excitation laser is parallel to the detection orientation [6, 40]. Herein, *I*, *I*_0_and θ are the measured SHG intensity, the maximum intensity, and the rotation angle of the incident laser polarization, respectively. To quantify the SHG intensity of 3R‐TaS_2_ compared to other 2D TMDs, a 3R‐MoS_2_ flake with similar thickness is used as a reference sample in this study. The SHG measurement of both 3R‐TaS_2_ and 3R‐MoS_2_ is conducted on a sapphire substrate. To ensure a consistent optical environment for comparison, the comparative SHG measurement with 3R‐MoS_2_ was carried out under the same experimental conditions at transmission mode (FW: 1040 nm, repetition rate: 80 MHz, pulse width: 100 fs, average power: 4 mW, laser beam diameter focused on sample: 7 µm). As shown in Figure 2d, the measured SHG response of a 77‐nm‐thick 3R‐TaS_2_ is stronger than the reference 3R‐MoS_2_ (79 nm), indicating the pronounced and robust SHG potential despite its metallic nature. To enable a more meaningful comparison between 3R‐TaS_2_ and MoS_2_, we further estimated the effective second‐order nonlinear susceptibility 𝜒^(2)^ of both 3R‐TaS_2_ and 3R‐MoS_2_, as discussed in Note S6. The extracted values are 1.27 × 10^2^ pm/V and 1.09 × 10^2^ pm/V for 3R‐MoS_2_ and 3R‐TaS_2_, respectively. Although the electronic structure of 3R‐TaS_2_ is metallic, its effective nonlinear susceptibility remains comparable to that of semiconducting 3R‐MoS_2_. This result indicates that strong second‐order nonlinear optical response can persist in metallic van der Waals materials.

### 2.2 Wavelength and Thickness Dependent SHG of 3R‐TaS_2_

**FIGURE 3:**
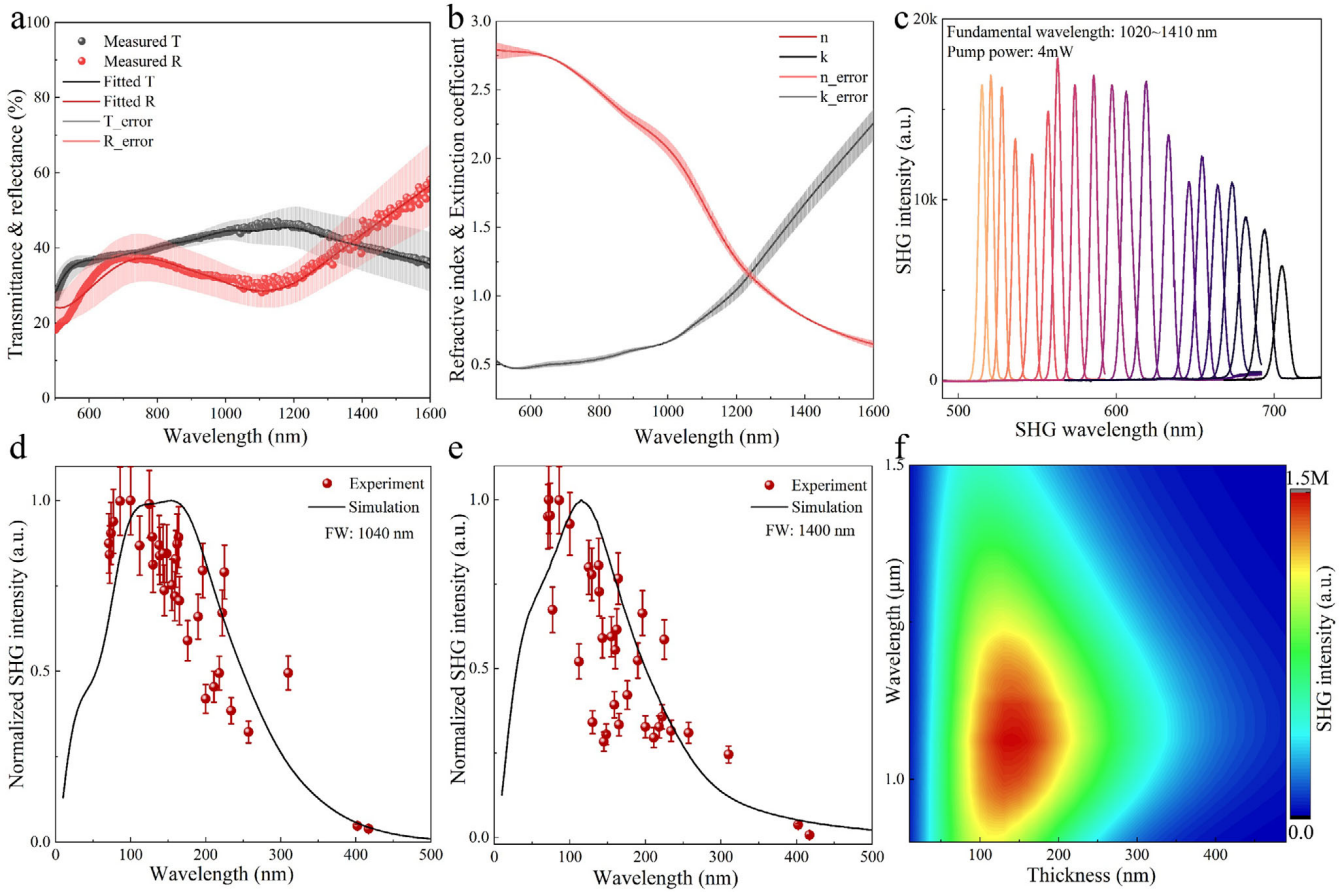
Wavelength and thickness‐dependent SHG. a) Transmittance (T, purple) and reflectance (R, orange) spectra of a specific 86‐nm‐thick 3R‐TaS_2_ flake on the sapphire substrate. Black and red solid lines are the fitted T and R spectra using the transfer matrix method (TMM) that parametrizes the dielectric function of 3R‐TaS_2_. b) Refractive index (*n*) and extinction coefficient (*k*) of the refractive index of bulk 3R‐TaS_2_ ($\tilde{n}=n+ik$). Here *n* and *k* are extracted from (a). c) Wavelength‐dependent SHG under the excitation wavelength from 1020 to 1410 nm. The thickness of the flake used for the wavelength‐dependent SHG measurement is 86 nm. d) Measured thickness‐dependent SHG intensity of 3R‐TaS_2_ (red dots) with respect to the calculated theoretical enhancement (black line) at FW and SH wavelengths of 1040 and 520 nm, respectively. e) Measured thickness‐dependent SHG intensity of 3R‐TaS_2_ (red dots) with respect to the calculated theoretical enhancement (black line) at FW and SH wavelengths of 1400 and 700 nm, respectively. The average pump power is kept constant at 4 mW, and the linear pump polarization is parallel to the armchair direction. f) Simulated results of the SHG response at various FWs and thicknesses through the COMSOL Multiphysics Wave optics modelling.

Reflectance R(λ) and transmittance T(λ) spectra were measured for a nominally 86 nm thick 3R‐TaS_2_ flake on a sapphire substrate, as shown in Figure 3a. The *R*(λ) and *T*(λ) of the air/film/sapphire/air structure are calculated through a standard transfer‐matrix method at normal incidence. The fitting details can be referred to Note S7. To probe the interaction between incident laser and SHG response of 3R‐TaS_2_, the full refractive‐index spectrum of an 86‐nm‐thick flake on a sapphire substrate has been investigated. The real and imaginary refractive index (*n* and *k*) are derived through the complex dielectric function from the measured reflectance and transmittance spectra in Figure 3a. The spectra of the complex dielectric function over the full measured wavelength range are plotted in Figure S6. The detailed derivation process of *n* and *k* is given in Note S7. Based on the sign of Re[ε] in Figure S6, we find that the material exhibits metallic‐like optical behavior only in the long‐wavelength region above 1237 nm, where Re[ε]<0, while in the spectral range from 500 to 1237 nm relevant to our SHG measurements, Re[ε] remains positive, indicating a dielectric‐like optical response. Notably, while DFT results indicate a metallic/semimetallic electronic structure, the experimentally extracted dielectric function shows that the real part of the permittivity remains positive within the visible spectral range relevant to the SHG measurements. Consequently, although free carriers are present, the optical response in this spectral region remains predominantly dielectric‐like rather than strongly metallic. This distinction can also help explain the strong and broadband SHG despite the metallic/semimetallic electronic structure of 3R‐TaS_2_ in this work. The extracted n and k in Figure 3b exhibit qualitatively similar spectral trends to those reported by Munkhbat et al., demonstrating the Drude‐like metallic behavior of the 3R‐TaS_2_ [41]. Although the crystal phase reported by Munkhbat et al. is 2H‐TaS_2_, both 2H and 3R‐TaS_2_ are metallic and share similar electronic structures near the Fermi level. Therefore, the Drude‐like response remains physically consistent. Quantitative differences in magnitude may arise from variations in crystal phase, sample thickness, or experimental configuration. SHG responses of 3R‐TaS_2_ for different pump wavelengths with an average pump power of 4 mW are illustrated in Figure 3c. It has been reported that the SHG response of a MoS_2_ flake is predominantly governed by excitonic effects, exhibiting strong dependence on the excitation wavelength and rapidly diminishing when the wavelength deviates from the fundamental resonance associated with the maximum SHG response [40, 42]. Remarkably, pronounced SHG signals are observed over a broad emission bandwidth (510–705 nm) in 3R‐TaS_2_, without being restricted to narrow resonance‐enhanced spectral regions, indicating a broadband nonlinear response distinct from the resonance‐dominated behavior observed in semiconducting 3R‐MoS_2_. Furthermore, the SHG response in the wavelength range from 1240 to 1410 nm slowly descends due to the metallicity of 3R‐TaS_2_. This trend is consistent with the transition from dielectric‐like optical behavior in the visible range to a stronger free‐carrier‐dominated response in the near‐infrared region. Overall, the observed broadband SHG in 3R‐TaS_2_ can be understood as a combined consequence of inversion‐symmetry breaking, dielectric‐like optical response in the visible range, free‐carrier‐induced damping, and broadened nonlinear resonance behavior associated with its metallic/semimetallic electronic structure. To exclude possible variations arising from the wavelength‐dependent response of the optical system, comparative SHG measurements were performed on a LiNbO_3_ reference thin film under identical experimental conditions. The distinct spectral evolution observed in LiNbO_3_ confirms that the broadband SHG response of 3R‐TaS_2_ is intrinsic to the material rather than originating from the measurement system. Details of LiNbO_3_ calibrations can be referred to Note S8 (Figure S7). Furthermore, the dependence of SHG intensity on the thickness of 3R‐TaS_2_ was systematically investigated. The measured SHG intensities for flakes with different thicknesses at FW of 1040 nm are summarized in Figure 3d together with the simulated trend calculated by the Wave Optics Module in COMSOL Multiphysics with a 3D model (black solid line). The simulation process can be referred to the Experimental Section. Although noticeable experimental fluctuations exist, particularly in the intermediate thickness range, the overall thickness‐dependent behavior is reasonably reproduced by the model. The experimental SHG fluctuations are mainly attributed to local variations in thickness, surface morphology, and the strong sensitivity of interference‐modulated SHG to phase accumulation and optical absorption in this thickness regime. Notably, with the consideration of free carriers, the fitting results of thickness‐dependent SHG do not show similar periodic oscillations observed in 3R‐MoS_2_ [10]. To investigate the role of metallicity in the SHG response of 3R‐TaS_2_, Figure 3e compares the thickness‐dependent SHG intensity under FW excitation at 1040 and 1400 nm. For both excitation wavelengths, the SHG intensity decreases with increasing flake thickness. This thickness‐dependent decay can be attributed to the increased optical absorption and propagation loss in thicker flakes, which attenuate the fundamental field and reduce the effective nonlinear polarization contributing to SHG emission. More importantly, the SHG intensity decays more rapidly at 1400 nm than at 1040 nm. This difference is closely related to the wavelength‐dependent optical response of 3R‐TaS_2_. As shown in Figure 3b, the extinction coefficient k increases significantly in the near‐infrared region, indicating stronger free‐carrier absorption and Drude‐like damping at longer wavelengths. Therefore, the fundamental field at 1400 nm experiences stronger attenuation inside thicker flakes, leading to a faster reduction of the effective nonlinear polarization and weaker SHG emission. In addition, enhanced free‐carrier damping can suppress coherent interference effects and smooth the thickness‐dependent SHG evolution. These results suggest that the thickness‐dependent SHG decay, particularly the faster suppression at 1400 nm, is closely associated with the metallic/semimetallic optical response of 3R‐TaS_2_. The simulation reproduces this overall trend reasonably well, which is plotted in Figure 3f.

### 2.3 Gold Enhanced SHG by Metallic Interface Interaction

**FIGURE 4:**
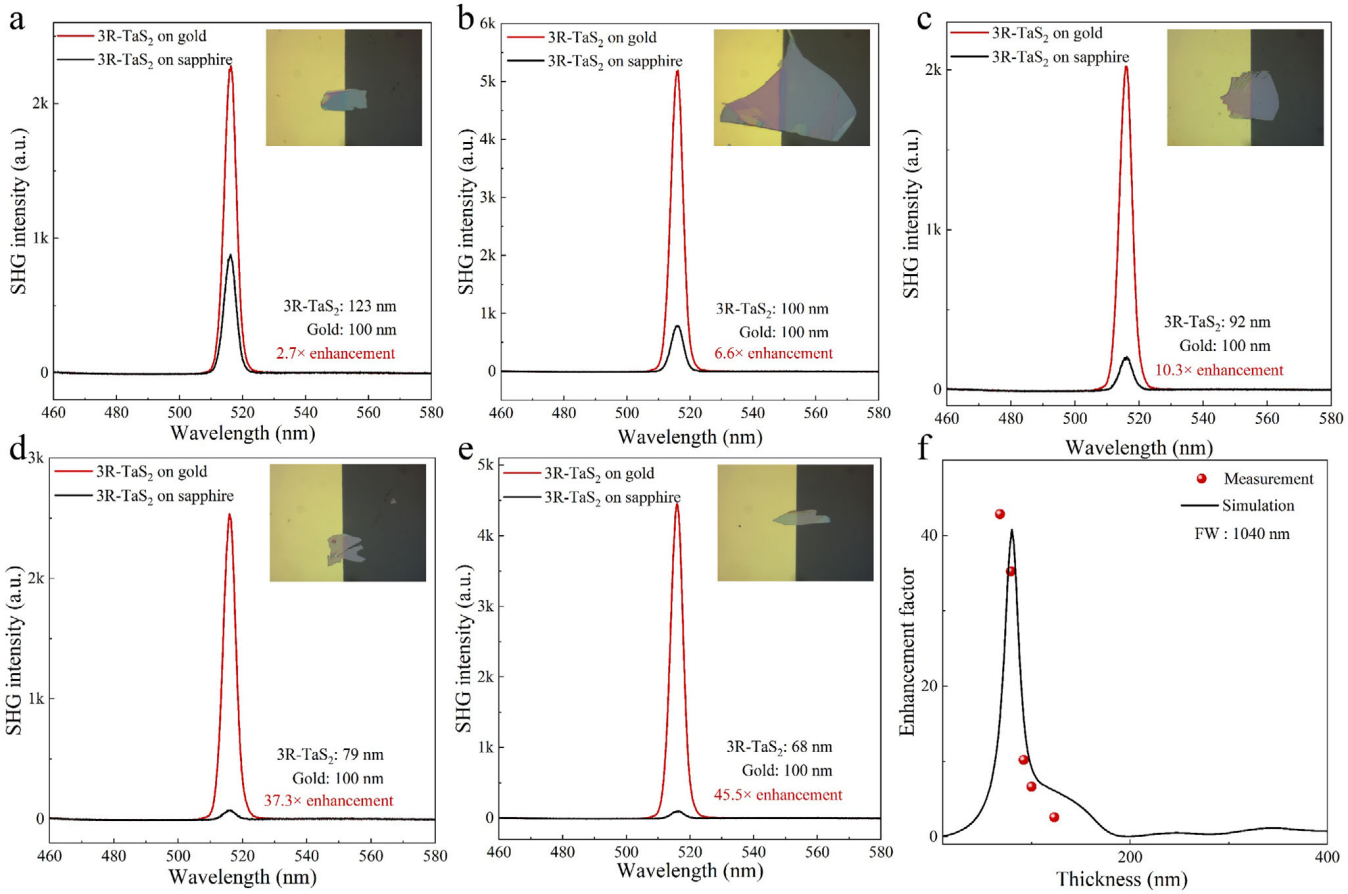
SHG intensity of 3R‐TaS_2_ enhanced by a gold surface. a‐e) SHG intensity of 3R‐TaS_2_ samples with various thicknesses of 123, 100, 92, 79, and 68 nm, respectively. The red lines are the SHG signals of 3R‐TaS_2_ measured on the gold surface, while the black lines are measured on the sapphire substrate. The enhancement factors are 2.7, 6.6, 10.3, 37.3, and 45.5, respectively. The FW is 1040 nm, and the average excitation power is 4 mW. f) Experimental enhancement factors as a function of thickness (red dots) with respect to the calculated theoretical enhancement (black line) by the COMSOL Multiphysics Wave optics modelling.

In order to further enhance the SHG response in such a 2D metallic nonlinear system, we have investigated the metallic interface interaction on the nonlinear behaviors of 3R‐TaS_2_. Lyu et al. recently demonstrated that the nonlinear conversion efficiency can be greatly enhanced by a gold substrate due to the large reflectivity of the gold surface [11]. The metal‐dielectric interface would cause strong SHG owing to the electric field discontinuity at the interface due to the huge permittivity contrast between metal and dielectric, while the SHG response would typically be suppressed due to the continuous electric field at a conventional metal‐metal interface [43]. Herein, we study the effect of a gold substrate on the SHG response of metallic 3R‐TaS_2_. Surprisingly, the SHG intensity of 3R‐TaS_2_ at the metallic interface is not quenched but rather enhanced. According to Figure 4a–e, regarding the measured SHG responses relative to those on sapphire substrates, we can observe that the enhancement factors on gold substrate vary significantly with sample thicknesses. The experimental trend of enhancement factors as a function of thickness can be reasonably well reproduced by calculation through the COMSOL Multiphysics Wave optics modelling, which is plotted in Figure 4f. The amplitude of SHG signals for 68 nm‐thick 3R‐TaS_2_ on a gold substrate is 45.5‐fold higher than that on sapphire substrates, while the amplification of the other samples with various thicknesses (123, 100, 92, and 79 nm) are 2.7, 6.6, 10.3, and 37.3 folds (Figure 4b–e), respectively. This trend suggests that the Au‐induced enhancement is not a simple bulk amplification effect but is mainly governed by the redistribution of the optical field near the metallic interface. Since the gold film is sufficiently thick (100 nm) and smooth, the metal surface does not provide the necessary conditions, such as a roughened surface or nanostructures, to support plasmonic effects [44, 45]. Thus, plasmonic effects at the 3R‐TaS_2_/gold interface can be excluded in this study. Instead, the localization of the electric field of the incident light and SH emission light would play an important role on the SH conversion [46]. Therefore, the intensity of SHG can be extracted by the formula as follows:

(1) $$I_{2\omega }^{\mathrm{ref}}\left(d\right)\propto I_{\omega }^{2}{\left|\int_{0}^{d}\chi^{\left(2\right)}F_{\omega }^{2}\left(z,d\right)G_{2\omega }\left(z,d\right)e^{i\Delta kz}\;dz\right|}^{2}$$

Here, *F*_ω_(*z*,*d*) is the local fundamental‐field factor, *G*_2ω_(*z*,*d*) is the outcoupling factor for SHG emitted toward the reflection side, and *e* ^*i*Δ*kz*^ describes the coherent phase accumulation of SHG generated at different depths.

The Au substrate significantly modifies the local electromagnetic field distribution near the 3R‐TaS_2_/gold interface, which is much larger than the 3R‐TaS_2_/sapphire interface $\left(E_{\omega }^{Au}>>E_{\omega }^{sapphire}\right)$. In particular, the reflected fundamental wave interferes with the incident field, leading to a strong enhancement of the electric field near the metal interface. Since the second‐harmonic polarization scales with the square of the local field $\left(P_{2\omega }^{\left(2\right)}\propto \varepsilon_{0}\chi^{\left(2\right)}E_{\omega }^{2}\right)$, this interfacial field enhancement can substantially increase the SHG generation. However, the enhanced field is mainly confined to the region close to the metal surface. The strongest enhancement observed around the 68 nm flake therefore likely reflects an optimal balance between the interfacial field enhancement and the effective nonlinear interaction volume. Such thickness‐dependent gold enhancement is consistent with previous studies of metal‐supported nonlinear 2D materials, where the metallic substrate redistributes the optical field and strongly modifies the effective nonlinear response [46]. It's worth noting that the enhancement factor of 45.5 folds in 3R‐TaS_2_ in our case is higher than that in insulating hBN and semiconducting MoS_2_ at the same thickness regime [11, 46]. The metallic electronic structure of 3R‐TaS_2_ may facilitate efficient nonlinear polarization under near‐infrared excitation. Compared with wide‐bandgap materials such as hBN or exciton‐dominated semiconductors such as MoS_2_, the broader electronic transitions in 3R‐TaS_2_ can support a stronger and more broadband nonlinear optical response. This intrinsic property, combined with the metal‐induced field enhancement at the Au interface, likely contributes to the exceptionally large SHG enhancement observed in the present system. At the metallic interface between 3R‐TaS_2_ and gold, the local optical field cannot form enhanced bulk effects as in dielectric h‐BN due to the strong free‐carrier screening and high absorptive nature (large extinction coefficient (*k*)). The absorption spectrum in Figure S8 provides evidence that 3R‐TaS_2_ exhibits a lossy optical response over the studied spectral range. Since the lossy optical response suppresses strong cavity‐like constructive interference inside the flake, the gold‐induced enhancement is mainly localized near the 3R‐TaS_2_/Au interface. Thicker flakes introduce a longer propagation path for both the incident fundamental field and the generated SHG signal. As a result, the fundamental field reaching the interface is attenuated, while the interface‐generated SHG can be partially reabsorbed during propagation through the flake. Therefore, the reduction of the enhancement factor with increasing thickness should be attributed to increased cumulative optical attenuation and the reduced relative contribution of the interfacial enhancement.

## 3 Conclusion

In summary, we systematically investigated the second‐order nonlinear optical response of metallic 3R‐TaS_2_ through wavelength‐, polarization‐, and thickness‐dependent SHG measurements. Remarkably, strong SHG is observed over a broad emission wavelength range of 510–705 nm despite the presence of free carriers that are generally expected to suppress nonlinear optical processes in metallic systems. Analysis of the optical constants and dielectric function reveals that the broadband SHG response is associated with the non‐centrosymmetric crystal structure, dielectric‐like optical response in the visible spectral range, and the absence of sharp resonance features. Furthermore, the SHG intensity can be effectively modulated by a reflective gold substrate. Thinner flakes exhibit stronger enhancement because the incident optical field can more efficiently reach the 3R‐TaS_2_/Au interface, while the generated SHG signal experiences reduced propagation loss and reabsorption. The thickness‐dependent behavior, together with the wavelength‐dependent optical response, indicates that free‐carrier‐induced damping and optical absorption play important roles in shaping the nonlinear optical properties of 3R‐TaS_2_. The enhanced SHG is therefore attributed primarily to interface‐mediated local‐field modulation and nonlinear optical contributions at the 3R‐TaS_2_/Au interface. More importantly, the extracted effective second‐order susceptibility of 3R‐TaS_2_ is comparable to that of MoS_2_ despite its metallic electronic structure. These results demonstrate that strong and broadband second‐order nonlinear optical responses can persist in metallic layered materials, extending the scope of nonlinear van der Waals materials beyond conventional semiconductors and insulators. This work provides new insights into the interplay between metallicity, optical response, and nonlinear light–matter interactions, while offering a promising platform for nonlinear photonics based on metallic layered crystals.

## 4 Experimental Section

### 4.1 Synthesis of 3R‐TaS_2_ Single Crystals

High‐quality 3R‐TaS_2_ single crystals were synthesized using a two‐step chemical vapor transport (CVT) method. Initially, 3R‐TaS_2_ polycrystalline powder was prepared as a precursor. High‐purity Ta powders (Macklin, 99.99%) and S powders (Macklin, 99.99%) were mixed in a molar ratio of 1:2.1 and placed into an evacuated quartz tube, which was then sealed. The mixture was heated to 950°C over a period of 10 h and maintained at this temperature for 50 h, and then quenched in iced‐water for voiding mixed phases. Subsequently, the 3R‐TaS_2_ polycrystalline powder was combined with iodide (Macklin, 99.99%) in a weight ratio of 1: 0.1, and sealed into a 14 cm quartz ampoule after evacuating to a pressure of less than 10^−4^ Torr. The reaction zone was then programmed at a higher temperature of 850°C with the growth zone at a lower temperature of 750°C for 15 days. Single crystals of 3R‐TaS_2_ were obtained from the cold zone. These crystals were found to be stable, showing no degradation or structural phase transitions when stored in air.

### 4.2 Sample Preparation

The as‐synthesized 3R‐TaS_2_ was exfoliated by scotch tape and a polydimethylsiloxane (PDMS) film. Before transferring the exfoliated samples, all sapphire substrates were pre‐treated by oxygen plasma for 10 min to remove potential contamination and improve the adhesion force between 3R‐TaS_2_ samples and the sapphire substrates. The measured flakes with different thicknesses and suitable lateral dimensions were first identified and selected through an optical microscope according to the interference colors. To prepare samples for gold‐enhanced measurement, gold pads with a lateral dimension of 1000 µm × 1000 µm and a thickness of 100 nm were fabricated on the sapphire substrates through ultraviolet (UV) photolithography (MLA 150 Maskless Aligner) and ebeam evaporation. Specific samples on PDMS are selected and dry transferred to the substrates with gold pads. Herein, the material was transferred with half overlapping the gold pad and half on the sapphire substrate, enabling comparative measurements for the same sample on different substrates. All measured samples in our work have a lateral size larger than 10 µm × 10 µm, which is large enough for the SHG measurement.

### 4.3 Material Characterization

Room‐temperature single‐crystal X‐ray diffraction (SXRD) measurements were conducted using a Bruker D8 single‐crystal X‐ray diffractometer with a Mo Kα radiation source (λ═ 0.71073 Å) at room temperature (Figure S9). Optical images of 3R‐TaS2 flakes were taken by an optical microscope (Nikon Eclipse LV150NL). Sample height profile and surface topography images were extracted through a scattering‐type scanning near‐field optical microscope (neaSNOM) conducted at tapping mode. The Raman spectrum was measured by a Raman spectrometer at the excitation wavelength of 532 nm. The excitation power was 11 mW, and the data acquisition duration was 60s.

### 4.4 SHG Measurement

For wavelength‐dependent and power‐dependent SHG, the SHG signals were collected from samples on the sapphire substrates at transmission mode through a home‐built SHG measurement setup (Figure S3). As illustrated in Figure S3, a Mai Tai fs pulse laser combined with an OPO (wavelength range: 1020–1410 nm, repetition rate: 80 MHz, and pulse width: 100 fs) was used as the fundamental pump source. The laser beam was focused onto the sample with a beam diameter of approximately 7 µm. The flip mirror before the 20× Mitutoyo Plan Apo near‐infrared (NIR) objective lens with a numerical aperture (NA) of 0.40 and the beam splitter were flipped down during the transmitted SHG measurements. The polarization angle of the incident laser was controlled by the half‐wave plate. The linearly polarized light went through the 20× near infrared (NIR) objective lens and focused on the samples. The SHG signal was collected by a 50× Mitutoyo Plan Apo near infrared (NIR) objective lens with an NA of 0.42, which was finally detected and analyzed by a spectrometer (Andor Kymera 328i) and a cooled silicon charge‐coupled device (CCD) camera (Andor iDus 420 CCD). It should be noted that no analyzer was used during the transmitted SHG measurement.

For polarization‐dependent SHG, a linear polarizer functioning as the analyzer was employed to analyze the polarization of the SHG signal (Figure S3). The incident polarization and linear polarizer were placed in a perpendicular configuration. The half‐wave plate was positioned in front of the laser source to ensure the polarization of the pump beam, while the analyzer was positioned in front of the detector to analyze the SHG polarization. During the polarization‐dependent SHG measurement, the analyzer was rotated synchronously with the incident polarization.

For gold‐enhanced SHG measurement, the SHG signals were collected from samples on the sapphire substrates at reflection mode through our home‐built SHG measurement setup (Figure S3). A 50× NIR objective lens (NA ═ 0.42) was used for both excitation and collection in the reflection‐mode SHG measurements performed on samples supported by sapphire/gold substrates. During the reflective SHG measurement, the flip mirror before the 20× objective lens and the beam splitter were flipped up. As shown in Figure S3, the linearly polarized laser with a fundamental wavelength (FW) of λ is redirected by the flip mirror, then deflected by 180° by two mirrors, and reaches the front side of the 3R‐TaS_2_ through the 50× objective lens. The reflective SHG signal with a wavelength of λ/2 is collected through the same 50× objective lens. The FW of the reflective SHG measurement is fixed at1040 nm. Herein, we used the same laser source as that in the transmitted SHG measurement. The schematic illustration of the reflective SHG setup can be referred to Note S3 (Figure S3).

The SHG mapping image was captured at reflection mode by Zeiss Confocal Microscopy equipped with a tunable Ti: Sapphire pulse laser (tunable wavelength range: 820‐1040 nm, pulse width: 150 fs, repetition rate: 80 MHz). The sample was measured under the 50× confocal objective lens (NA═ 0.85) at reflection mode with an excitation wavelength of 1020 nm.

### 4.5 Linear Optical Transmission and Reflection Measurements

To obtain the absorption spectra and extract the complex refractive index, 3R‐TaS_2_ flakes on the transparent substrates are exposed to white light generated by an OSL2IR Fiber illuminator from Thorlabs. Herein, sapphire substrates are used due to their high transparency in the excitation range of the pump light to ensure accuracy of data collection in transmission measurements. The linear measurements were performed using the same experimental setup as SHG measurements, with only slight modification. The laser source in the SHG setup was replaced by a broadband illuminator (Thorlabs OSL2IR), and the detectors were fibre‐coupled CCD and InGaAs (NIRQUEST+2.2‐25., SR‐6VIS400‐25), which can collect signals from the visible to near‐infrared wavelength range. The measurements were performed using two 50× objective lenses (NA ═0.42), which are designed to excite sample and collect results respectively. Additionally, a gold‐coated mirror is used to calibrate the pump light intensity for the reflection measurement.

### 4.6 Second Harmonic Simulation

The SHG efficiency of the thin nonlinear layer was calculated using the Wave Optics Module in COMSOL Multiphysics with a 3D model. The simulation followed the frequency‐domain SHG framework. Two Electromagnetic Waves, Frequency Domain interfaces were used in the same geometry: one was assigned to the fundamental frequency ω, and the other was assigned to the second‐harmonic frequency 2ω.

In the fundamental‐frequency interface, the incident light was defined as a normally incident plane wave, and the electric field distribution E(ω) inside the nonlinear layer was solved. In the second‐harmonic interface, a Domain Polarization node was added to the nonlinear material domain. The second‐order nonlinear polarization was used as the source term for the 2ω field:

$$P_{2\omega }^{\left(2\right)}\propto \varepsilon_{0}\chi^{\left(2\right)}E_{\omega }^{2}$$

Here, χ^(2)^ was directly assigned in units of pm/V as the nonlinear tensor matrix according to the crystal symmetry and orientation of the material. The electric‐field components obtained from the fundamental‐frequency simulation were used to calculate the nonlinear polarization components P_x_, P_y_, and P_z_ for the second‐harmonic simulation.

## Conflicts of Interest

The authors declare no conflict of interest.

## Supporting information




**Supporting File**: smll74789‐sup‐0001‐SuppMat.docx.

## Data Availability

The data that support the findings of this study are available from the corresponding author upon reasonable request.
