# Supplementary material for: Broadband and Enhanced Second‐Harmonic Generation in Metallic 3R‐TaS2
Source: Small. 2026 Aug 14;22(53):e13532. doi: 10.1002/smll.202513532 (PMC13592414; doi:10.1002/smll.202513532)
Supplement: Supplementary file 1 [file SMLL-22-e13532-s001.docx]

**Broadband and Enhanced Second-Harmonic Generation in Metallic 3R-TaS_2_**

Ruo-Si Chen^1†^, Chuanyu Wang^1†^, [Xiangqi Liu](https://arxiv.org/search/cond-mat?searchtype=author&query=Liu,+X)^2†^, Zhuoyuan Lu^1^, Shuyao Qiu^1^, Boqing Liu^1^, Tieyu Lü^3^, Shi-Rui Zhang^4^, [Yanfeng Guo](https://arxiv.org/search/cond-mat?searchtype=author&query=Guo,+Y)^2^*, Yuerui Lu^1^*

^1^School of Engineering, College of Systems and Society, Australian National University, Canberra, ACT, 2602, Australia

^2^ShanghaiTech Laboratory for Topological Physics, ShanghaiTech University, Shanghai, 201210, China

^3^Department of Physics and Institute of Theoretical Physics and Astrophysics, Xiamen University, Xiamen, China

^4^School of Physics, Department of Electronic Materials Engineering, Australian National University, Canberra, ACT, 2602, Australia

* Corresponding Authors

^†^ Co-first Authors

Email: [yuerui.lu@anu.edu.au](mailto:yuerui.lu@anu.edu.au); [guoyf@shanghaitech.edu.cn](mailto:guoyf@shanghaitech.edu.cn)

**Supplementary Note 1: Sample Characterization**

Herein, the excitation wavelength of the Raman spectroscopy is 532 nm, and the excitation power is 11 mW. The Raman spectra of the SiO_2_/Si substrate (black solid line) and 3R-TaS_2_ (red solid line) are obtained after an acquisition duration of 2 min. The $E_{2g}^{1}$ peak at 291 cm^-1^ is originated from the in-plane resonance of Ta and S atoms, and the $A_{1g}$ peak at 395 cm^-1^ is caused by the out-of-plane resonance of S atoms. The overlap of Raman peak between the 3R-TaS_2_ (291 cm^-1^) and the substrate (303 cm^-1^) contributes to the broad peak width of $E_{2g}^{1}$ peak.

**Figure S1.** Raman spectrum of 3R-TaS_2_ on 285-nm-thick SiO_2_/Si substrate.

**Supplementary Note 2: Demonstration of Metallic Properties of 3R-TaS_2_**

**Figure S2.** a) The band structure of 3R-TaS_2_. b) Density of states of 3R-TaS_2_.

**Supplementary Note 3: Experimental Setup**

**Figure S3.** Schematic illustration of the experimental setup for the SHG measurement (transmission and reflection modes).

**Supplementary Note 4: Cycling Test of 3R-TaS_2_**

**Figure S4.** Cycling test of a 3R-TaS_2_ with thickness of 86 nm. No irreversible damage or phase transition occurred during the measurement. The FW is 1040 nm, and the average excitation power is 5 mW.

**Supplementary Note 5: SHG Stability Measurement for Different Period of Time**

**Figure S5**. a-c) Long-term SHG stability measurements on three 3R-TaS₂ samples with the thicknesses of (a) 146 nm (a), 115 nm (b), and 46 nm (c) after exposure to ambient environment for different periods of time.

**Supplementary Note 6: Estimation of the Effective Second-Order Nonlinear Susceptibility**

The effective second-order nonlinear susceptibilities of 3R-MoS_2_ and 3R-TaS_2_ flakes were estimated by using an 600nm x-cut LiNbO_3_ thin film as the reference. All samples were measured in the same transmission SHG setup under identical optical alignment, collection geometry, detector settings, and excitation wavelength of 1040 nm. The 3R-MoS_2_ and 3R-TaS_2_ flakes were placed on 400-um-thick sapphire substrates.

The starting expression follows the thickness-dependent SHG conversion efficiency used in the Supplementary Information of Xu et al.^1^ In their transfer-matrix treatment, the pump transmission through the nonlinear slab and the finite phase-matching length are combined as

$\frac{I_{2\omega}}{I_{\omega}} \propto T_{\omega}\left( h \right)\left| \chi^{\left( 2 \right)} \right|^{2} \frac{I_{\omega}}{\Delta k^{2}} \sin^{2} \left( \frac{\Delta k h}{2} \right)$	(1)

Since the measured SHG counts are proportional to the emitted second-harmonic intensity and the incident fundamental intensity is proportional to the pump power, this expression can be rearranged into a relative-count form:

$C_{2\omega}\propto\left| \chi^{\left( 2 \right)} \right|^{2}P_{\omega}^{2}T_{\omega}\left( h \right)\frac{\sin^{2} \left( \Delta kh/2 \right)}{\Delta k^{2}}$	(2)

For the present samples, a directly calculated optical factor was used to collect the thickness-dependent buildup, absorption, Fresnel transmission, and local-field terms:

$C_{2\omega,j}=AP_{\omega,j}^{2}\left| \chi_{\text{eff},j}^{\left( 2 \right)} \right|^{2}F_{\text{opt},j}$	(3)

where A is a setup-dependent proportionality constant that is common to all samples. Therefore, using LiNbO_3_ as the reference gives

$\chi_{\text{eff},s}^{\left( 2 \right)}=\chi_{\text{LN}}^{\left( 2 \right)}\sqrt{\frac{C_{s}}{C_{\text{LN}}}}\frac{P_{\text{LN}}}{P_{s}}\sqrt{\frac{F_{\text{opt,LN}}}{F_{\text{opt},s}}}$	(4)

Here C is the measured SHG count, P is the incident pump power, and F_opt_ is calculated from the film optical constants, thickness, Fresnel coefficients, and coherent SHG buildup inside the film. This is the same relative-calibration logic as the source expression above, but F_opt_ is evaluated for the actual air/nonlinear-film/sapphire/air geometry instead of approximating the coherent buildup by h^2^.

The layer model air/nonlinear-film/sapphire/air used for the linear-optical analysis: layer 0 is the incident air medium, layer 1 is the coherent nonlinear thin film, layer 2 is the thick sapphire substrate, and layer 3 is the exit air medium at the backside of the substrate. The nonlinear film is treated coherently, while the 400-um sapphire substrate is treated incoherently. Therefore, the phase accumulated inside the sapphire substrate is not used as a coherent SHG phase term.

The complex refractive index is written as

$N_{\lambda,j}=n_{\lambda,j}+i\kappa_{\lambda,j}$	(5)

where κ is the extinction coefficient. The corresponding complex wavevectors at the fundamental and second-harmonic wavelengths are

$K_{\omega,j}=\frac{2\pi}{\lambda_{\omega}}N_{\omega,j} K_{2\omega,j}=\frac{2\pi}{\lambda_{2\omega}}N_{2\omega,j}$	(6)

At normal incidence, the Fresnel coefficients between layers a and b are

$r_{\mathrm{ab}}^{\left( \lambda,j \right)}=\frac{N_{a}^{\left( \lambda\right)}N_{b}^{\left( \lambda\right)}}{N_{a}^{\left( \lambda\right)}+N_{b}^{\left( \lambda\right)}} t_{\mathrm{ab}}^{\left( \lambda,j \right)}=\frac{2N_{a}^{\left( \lambda\right)}}{N_{a}^{\left( \lambda\right)}+N_{b}^{\left( \lambda\right)}}$	(7)

The fundamental field inside the coherent nonlinear film is written as

$E_{\omega,j}\left( z \right)=E_{\omega,j}^{+}\exp\left( iK_{\omega,j}z \right)+E_{\omega,j}^{-}\exp\left( -iK_{\omega,j}z \right)$	(8)

with the forward and backward amplitudes calculated for the coherent air/film/sapphire front stack:

$E_{\omega,j}^{+}=\frac{t_{01}^{\left( \omega,j \right)}}{1+r_{01}^{\left( \omega,j \right)}r_{12}^{\left( \omega,j \right)}\exp\left( 2iK_{\omega,j}h_{j} \right)} E_{\omega,j}^{-}=E_{\omega,j}^{+}r_{12}^{\left( \omega,j \right)}\exp\left( 2iK_{\omega,j}h_{j} \right)$	(9)

The forward-transmitted SHG amplitude is obtained by integrating the nonlinear polarization generated throughout the film, including propagation and absorption of the generated 520 nm field before it reaches the film/sapphire interface:

$A_{2\omega,j}\propto t_{12}^{\left( 2\omega,j \right)}t_{23}^{\left( 2\omega\right)}\int_{0}^{h_{j}} \left[ E_{\omega,j}\left( z \right) \right]^{2}\exp\left[ iK_{2\omega,j}\left( h_{j}-z \right) \right]\mathrm{dz}$	(10)

Thus the optical correction factor used for the relative calibration is

$F_{\text{opt},j}=\left| t_{12}^{\left( 2\omega,j \right)}t_{23}^{\left( 2\omega\right)}\int_{0}^{h_{j}} \left[ E_{\omega,j}\left( z \right) \right]^{2}\exp\left[ iK_{2\omega,j}\left( h_{j}-z \right) \right]\mathrm{dz} \right|^{2}$	(11)

Only ratios of F_opt_ are used in the final susceptibility calculation, so the absolute normalization of F_opt_ is arbitrary as long as the same convention is applied to LiNbO_3_, 3R-MoS_2_, and 3R-TaS_2_. The directly evaluated F_opt_ values are listed in Table S1.

The nonlinear coefficient of congruent LiNbO_3_ was referenced from Shoji et al.^2^ The reported wavelength-dependent d_33_ values were linearly extracted to the experimental fundamental wavelength of 1040 nm, giving

$d_{33}^{\text{LN}}\left( 1040 \text{nm} \right)=25.32 \text{pm}\text{/}\text{V}$	(12)

Using the convention

$\chi^{\left( 2 \right)}=2d$	(13)

the reference susceptibility is

$\chi_{\text{LN}}^{\left( 2 \right)}=2d_{33}^{\text{LN}}=50.64 \text{pm}\text{/}\text{V}$	(14)

The optical constants used at the fundamental and second-harmonic wavelengths are listed in **Table S1**.

**Table S1.** The optical constants used at the fundamental and second-harmonic wavelengths.

| **Sample** | **h** | **P_ω_** | **SHG counts** | **N (1040 nm)** | **N (520 nm)** | **F_opt_ (a.u.)** | **χ_eff_^(2)^** | **nk source** |
| --- | --- | --- | --- | --- | --- | --- | --- | --- |
| x-cut LiNbO_3_ | 600 nm | 0.5 mW | 11335 | 2.2336 + 0i | 2.3293 + 0i | 8.9923 × 10⁴ | 50.64 pm/V | ^[3]^ |
| 3R-MoS_2_ | 79 nm | 4 mW | 19897 | 3.1477 + 0.04944i | 3.7652 + 0.67601i | 3.9237 × 10² | 126.96 pm/V | ^[4]^ |
| 3R-TaS_2_ | 77 nm | 4 mW | 24385 | 1.94379 + 0.72415i | 2.78591 + 0.49598i | 6.5561 × 10² | 108.73 pm/V | This work |

Substituting the measured counts, pump powers, and calculated optical factors into the relative calibration equation gives

$\chi_{\text{eff}}^{\left( 2 \right)}\left( \text{Mo}\text{S}_{\text{2}} \right)=126.96 \text{pm}\text{/}\text{V}\approx1.27\times{10}^{2} \text{pm}\text{/}\text{V}$	(15)

$\chi_{\text{eff}}^{\left( 2 \right)}\left( \text{Ta}\text{S}_{\text{2}} \right)=108.73 \text{pm}\text{/}\text{V}\approx1.09\times{10}^{2} \text{pm}\text{/}\text{V}$	(16)

These values should be interpreted as effective film nonlinear susceptibilities under the stated optical model. They include the effects of finite film thickness, optical interference, absorption, Fresnel transmission, stacking-dependent nonlinear response, and possible surface/interface contributions. They should therefore not be interpreted as isolated intrinsic bulk tensor elements.

**Supplementary Note 7: Measurement and extraction of effective optical constants**

The effective optical constants of the 3R-TaS_2_ flake were obtained by fitting the measured transmittance (T) and reflectance (R) spectra using a transfer matrix method (TMM). Instead of using a fully free oscillator parameterization as the final method, we used reported 2H-TaS_2_ optical constants as a reference spectrum and allowed smooth corrections to n and k. This choice is based on the fact that 2H and 3R polytypes of layered TMDs mainly differ in the stacking sequence of similar X-M-X layers, so their linear optical constants are generally expected to be closed, whereas symmetry-dependent nonlinear responses can differ more strongly. Similar behavior has been discussed for layered TMD polytypes and for 2H/3R MoS_2_, where the overall electronic and optoelectronic structures are closely related.^5-8^

The reference spectrum is therefore used only as a fitting starting point and regularization reference. The final n and k are not assumed to be identical to the reference values, but are obtained after fitting the measured T and R spectra.

The 3R-TaS_2_ flakes were placed on sapphire substrates for the linear optical measurement. The transmittance spectrum was measured by collecting the transmitted signal from the sample/sapphire region and normalizing it to the transmitted signal from a nearby bare sapphire region under the same alignment. After background subtraction,

$T_{\text{meas}}\left( \lambda\right)=\frac{I_{T,\text{sample}}\left( \lambda\right)-I_{T,\text{bg}}\left( \lambda\right)}{I_{T,\text{sapphire}}\left( \lambda\right)-I_{T,\text{bg}}\left( \lambda\right)}$	(17)

The reflectance spectrum was measured from the same sample region and calibrated using a gold reference measured under the same illumination and collection geometry. After background subtraction,

$R_{\text{meas}}\left( \lambda\right)=\frac{I_{R,\text{sample}}\left( \lambda\right)-I_{R,\text{bg}}\left( \lambda\right)}{I_{R,\text{Au}}\left( \lambda\right)-I_{R,\text{bg}}\left( \lambda\right)}R_{\text{Au}}\left( \lambda\right)$	(18)

In this air/nonlinear-film/sapphire/air model, layer 0 is the incident air medium, layer 1 is the coherent 3R-TaS_2_ thin film, layer 2 is the thick sapphire substrate, and layer 3 is the exit air medium at the backside of the substrate. Layers 0 and 3 are treated as semi-infinite media. The thickness of 3R-TaS_2_ film is $d_{1}$, and the sapphire substrate has a physical thickness of approximately $400\sim\mu\text{m}$. The 3R-TaS_2_ layer was treated as a coherent film, while the sapphire substrate was treated incoherently because the phase accumulated in the substrate is randomized over multiple internal passes. The complex refractive index of the 3R-TaS_2_ film is

$N\left( \lambda\right)=n\left( \lambda\right)+ik\left( \lambda\right)$	(19)

For an incident angle $\theta$ and polarization $\sigma$ = s or p, the optical admittance is

$q_{j}^{\left( s \right)}=N_{j}\cos\theta_{j} q_{j}^{\left( p \right)}=\frac{\cos\theta_{j}}{N_{j}}$	(20)

where$\theta_{j}$ is obtained from Snell’s law. The Fresnel coefficients between layers i and j are

$r_{\mathrm{ij}}=\frac{q_{i}-q_{j}}{q_{i}+q_{j}} t_{\mathrm{ij}}=\frac{2q_{i}}{q_{i}+q_{j}}$	(21)

For the coherent front stack air/3R-TaS_2_/sapphire, the phase accumulated in the 3R-TaS_2_ film is

$\beta=\frac{2\pi N_{1}d_{1}\cos\theta_{1}}{\lambda}$	(22)

and the effective reflection and transmission amplitudes are

$r_{02}=\frac{r_{01}+r_{12}\exp\left( 2i\beta\right)}{1+r_{01}r_{12}\exp\left( 2i\beta\right)} t_{02}=\frac{t_{01}t_{12}\exp\left( i\beta\right)}{1+r_{01}r_{12}\exp\left( 2i\beta\right)}$	(23)

The corresponding intensity quantities are

$R_{02}=\left| r_{02} \right|^{2} T_{02}=Re\left( \frac{q_{2}}{q_{0}} \right)\left| t_{02} \right|^{2}$	(24)

The reverse-direction quantities $R_{20}$ and $T_{20}$ for sapphire/3R-TaS_2_/air were calculated in the same way. The sapphire/air backside interface gives $R_{23}$ and $T_{23}$. Including incoherent backside reflections from the thick sapphire substrate,

$T_{\mathrm{sample}}=\frac{T_{02}T_{23}}{1-R_{20}R_{23}} R_{\mathrm{sample}}=R_{02}+\frac{T_{02}R_{23}T_{20}}{1-R_{20}R_{23}}$	(25)

The modeled T and R were averaged over the collection cone with $\theta_{\max}=\sin^{-1} \left( \text{NA} \right)$, using $\text{NA}=0.42$ and equal weighting of s and p\ polarizations:

$\left\langle T \right\rangle=\frac{1}{2}\sum_{\sigma=s,p} \frac{\int_{0}^{\theta_{max}} T_{\sigma}\left( \theta\right)W\left( \theta\right)d\theta}{\int_{0}^{\theta_{max}} W\left( \theta\right)d\theta} \left\langle R \right\rangle=\frac{1}{2}\sum_{\sigma=s,p} \frac{\int_{0}^{\theta_{max}} R_{\sigma}\left( \theta\right)W\left( \theta\right)d\theta}{\int_{0}^{\theta_{max}} W\left( \theta\right)d\theta}$	(26)

The fitted n and k were written as smooth corrections to the reference 2H-TaS_2_ optical constants:

$n\left( \lambda\right)=\max\left[ n_{\text{ref}}\left( \lambda\right)+\Delta n\left( \lambda\right), 0.02 \right] k\left( \lambda\right)=\max\left[ k_{\text{ref}}\left( \lambda\right)+\Delta k\left( \lambda\right), {10}^{-8} \right]$	(27)

Here $n_{\mathrm{ref}}$ and $k_{\mathrm{ref}}$ are the reference 2H-TaS_2_ optical constants, and $\Delta n$ and $\Delta k$ are fitted correction functions. The correction functions were described by piecewise cubic Hermite interpolation (PCHIP) through seven control points uniformly distributed over the fitted wavelength range. This allows the fitted 3R-TaS_2_ optical constants to deviate from the reference spectrum while avoiding unphysical oscillations between neighboring wavelengths. The lower bounds of $0.02$ for n and ${10}^{-8}$ for k were only used as numerical constraints to prevent unphysical negative refractive index or extinction coefficient values during the interpolation-based fitting; they do not represent additional fitting parameters or physical assumptions.

Small residual calibration differences in the T and R channels were included by scale and offset terms:

$T_{\mathrm{fit}}\left( \lambda\right)=a_{T}\left\langle T_{\mathrm{model}}\left( \lambda\right) \right\rangle+b_{T} R_{\mathrm{fit}}\left( \lambda\right)=a_{R}\left\langle R_{\mathrm{model}}\left( \lambda\right) \right\rangle+b_{R}$	(28)

The fitted parameters used for the accepted single-nk solution are summarized in **Table S2**.

**Table S2.** Parameters used in the accepted source-referenced single-nk fit. The seven correction values are the PCHIP control-point values added to the reference n and k spectra.

| **Parameter** | **Value** | **Description** |
| --- | --- | --- |
| $d_{\mathrm{Ta}S_{2}}$ | 86 nm | TaS2 thickness used for the center TMM solution |
| NA | 0.42 | Numerical aperture used for angular averaging |
| $\Delta n$ control points | -0.5376, 0.0497, 0.2825, 0.5722, 0.2789, -0.0017, -0.2211 | Seven PCHIP correction values for n |
| $\Delta k$ control points | -0.0446, 0.1634, 0.1290, -0.1281, -0.6142, -0.8527, -1.0154 | Seven PCHIP correction values for k |
| T/R calibration | $a_{T}=1.019599; b_{T}=0.001115;$  $a_{R}=1.264209; b_{R}=0.008639$ | Scale and offset factors applied to modeled T and R |

The complex dielectric function was calculated from the fitted n and k as

$\varepsilon\left( \lambda\right)=\varepsilon_{1}\left( \lambda\right)+i\varepsilon_{2}\left( \lambda\right)$	(29)

$\varepsilon_{1}\left( \lambda\right)=n^{2}\left( \lambda\right)-k^{2}\left( \lambda\right)$	(30)

$\varepsilon_{2}\left( \lambda\right)=2n\left( \lambda\right)k\left( \lambda\right)$	(31)

Herein, $\varepsilon_{1}\left( \lambda\right)$ and $\varepsilon_{2}\left( \lambda\right)$ refer to Re[$\varepsilon$] and Im[$\varepsilon$], respectively. The metallic-like optical response was evaluated from the sign of $\varepsilon_{1}$ rather than from the relative magnitudes of n and k.

**Figure S6.** Permittivity of 3R-TaS_2_.

The uncertainty bands for T, R, n, k, $\varepsilon_{1}$, and $\varepsilon_{2}$ were estimated using a fixed-center Monte Carlo propagation. The accepted single-nk solution was kept as the center line. A total of 500 Monte Carlo samples were generated by varying the TaS₂ thickness with $d\sim\mathcal{N}\left( 86\sim\text{nm},5\sim\text{nm} \right)$ and the reflectance scale factor with $a_{R}\mathcal{\sim N}\left( a_{R,0},0.10 \right)$, while keeping NA=0.42. For each sampled thickness, the correction from the reference n/k spectrum was rescaled by $\alpha=d_{0}/d$ to approximate the main thickness/nk correlation, and the TMM calculation was repeated.

**Supplementary Note 8: Wavelength-dependent System-response Calibration using LiNbO_3_ Reference**

To remove the wavelength-dependent response of the optical system, we used a LiNbO_3_ reference sample as an external calibration standard. The measured wavelength-dependent SHG signal from LiNbO_3_ contains both the intrinsic wavelength dependence of LiNbO_3_ and the wavelength-dependent response of the experimental system, including the excitation path, collection optics, filters, spectrometer/grating efficiency, and detector sensitivity. Therefore, by comparing the measured LiNbO_3_ SHG spectrum with the modelled LiNbO_3_ SHG response, we extracted a wavelength-dependent system response factor and applied it to calibrate the wavelength-dependent SHG data of 3R-TaS_2_.

Here, the wavelength variable λ denotes the fundamental excitation wavelength, and the SHG signal is detected at λ/2. For the reference LiNbO_3_, the measured SHG signal can be written as:

$I_{\mathrm{LN}}^{\mathrm{meas}}\left( \lambda\right)=C_{\mathrm{sys}}\left( \lambda\right)I_{\mathrm{LN}}^{\mathrm{model}}\left( \lambda\right)$	(32)

where $I_{\mathrm{LN}}^{\mathrm{meas}}\left( \lambda\right)$ is the experimentally measured wavelength-dependent SHG intensity of LiNbO_3_, $I_{\mathrm{LN}}^{\mathrm{model}}\left( \lambda\right)$ is the modelled LiNbO_3_ SHG response, and $C_{\mathrm{sys}}\left( \lambda\right)$ is the wavelength-dependent response of the experimental system.

The relative system response factor was then obtained as

$C_{\mathrm{rel}}\left( \lambda\right)=\frac{I_{\mathrm{LN}}^{\mathrm{meas}}\left( \lambda\right)/I_{\mathrm{LN}}^{\mathrm{model}}\left( \lambda\right)}{I_{\mathrm{LN}}^{\mathrm{meas}}\left( \lambda_{0} \right)/I_{\mathrm{LN}}^{\mathrm{model}}\left( \lambda_{0} \right)}$	(33)

where $\lambda_{0}$ is a selected reference wavelength.

The measured TaS_2_ wavelength-dependent SHG signal was then calibrated by dividing out this relative system response:

$I_{\mathrm{Ta}S_{2}}^{\mathrm{cali}}\left( \lambda\right)=\frac{I_{\mathrm{Ta}S_{2}}^{\mathrm{meas}}\left( \lambda\right)}{C_{\mathrm{rel}}\left( \lambda\right)}$	(34)

**Figure S7.** Measured and simulated wavelength-dependent SHG of LiNbO_3_ for calibration.

**Supplementary Note 9: Absorption Spectrum of 3R-TaS_2_**

**Figure S8.** Absorption spectrum (A) of the 86 nm thick 3R-TaS_2_ flake on sapphire substrate. Black solid line is the fitted A using the transfer matrix method (TMM) that parametrizes the dielectric function of 3R-TaS_2_. The absorption (A) spectrum is calculated from the measured transmittance (T) and reflectance (R) spectra using A=1−T−R.

**Supplementary Note 10: Single-Crystal X-ray Diffraction (SXRD) Measurements**

**Figure S9**. a-c) Images of single-crystal X-ray diffraction (SXRD) of 3R-TaS_2_. (a)-(c) are collected in reciprocal space along the (0*kl*), (*h*0*l*), and (*hk*0) planes, respectively. a= b= 3.33 Å, c= 18.07 Å.

**References**

1.	X. Xu, C. Trovatello, F. Mooshammer, Y. Shao, S. Zhang, K. Yao, D. N. Basov, G. Cerullo and P. J. Schuck, *Nat. Photonics*, 2022, **16**, 698-706.

2.	I. Shoji, T. Kondo, A. Kitamoto, M. Shirane and R. Ito, *J. Opt. Soc. Am. B*, 1997, **14**, 2268-2294.

3.	D. E. Zelmon, D. L. Small and D. Jundt, *J. Opt. Soc. Am. B*, 1997, **14**, 3319-3322.

4.	K. M. Islam, R. Synowicki, T. Ismael, I. Oguntoye, N. Grinalds and M. D. Escarra, *Adv. Photonics Res.*, 2021, **2**, 2000180.

5.	B. Munkhbat, P. Wrobel, T. J. Antosiewicz and T. O. Shegai, *ACS Photonics*, 2022, **9**, 2398-2407.

6.	C. Witteveen, K. Górnicka, J. Chang, M. Månsson, T. Klimczuk and F. O. von Rohr, *Dalton Trans.*, 2021, **50**, 3216-3223.

7.	R. M. Arif Khalil, F. Hussain, A. Manzoor Rana, M. Imran and G. Murtaza, *Physica E Low Dimens. Syst. Nanostruct.*, 2019, **106**, 338-345.

8.	S. S. Coutinho, M. S. Tavares, C. A. Barboza, N. F. Frazão, E. Moreira and D. L. Azevedo, *J. Phys. Chem. Solids.*, 2017, **111**, 25-33.
